# Expanding access to maternal, newborn and primary healthcare services through private-community-government partnership clinic models in rural Kenya: the Ubuntu-Afya kiosk model

**DOI:** 10.1186/s12913-019-4759-9

**Published:** 2019-11-29

**Authors:** Hellen Gatakaa, Elizabeth Ombech, Rogers Omondi, James Otiato, Vincent Waringa, Gordon Okomo, Richard Muga, Moses Ndiritu, Samson Gwer

**Affiliations:** 1 Research and Evidence Programme, Afya Research Africa, No. 12 Mai Mahiu Road, P.O. Box 20880-00202, Nairobi, Kenya; 2 Department of Health, Homa Bay County, Homa Bay, Kenya; 3 School of Medicine, Kenyatta University, Nairobi, Kenya

**Keywords:** Maternal and newborn health, Primary healthcare, Public-private partnership

## Abstract

**Background:**

Fifteen counties contribute 98.7% of the maternal and newborn morbidity and mortality in Kenya. The dismal maternal and newborn (MNH) outcomes in these settings are mostly attributable to limited access to skilled MNH services. Public health services are stretched and limited in reach, and many social programmes are not sustainably designed. We implemented a network of 16 self-sustaining community medical centres (Ubuntu-Afya Kiosks) in Homa Bay County, to facilitate access to MNH and other primary health services. We investigated the effect of these centres on MNH access indicators over a 2-year period of initial implementation.

**Methods:**

We conducted a baseline and end-line survey in June 2016 and May 2018 respectively, in 10 community health units (CHU) served by Ubuntu-Afya Kiosks. We targeted women of child bearing age, ensuring equal sample across the 10 CHUs. The surveys were powered to detect a 10% increase in the proportion of women who deliver under a skilled birth attendant from a perceived baseline of 55%. Background characteristics of the respondents were compared using Fisher’s exact test for the categorical data. STATA ‘svy’ commands were used to calculate confidence intervals for the proportions taking into account the clustering within CHU.

**Results:**

The coverage of antenatal care during previous pregnancy was 99% at end-line compared to 81% at baseline. Seventy one percent of mothers attended at least four antenatal care visits, compared to 64% at baseline. The proportion of women who delivered under a skilled birth attendant during previous pregnancy was higher at end-line (90%) compared to baseline (85%). There was an increase in the proportion of women who had their newborns examined within 2 day of delivery from 74 to 92% at end-line. A considerable proportion of the respondents visited private clinics at end-line (31%) compared to 3% at baseline.

**Conclusions:**

Ubuntu-Afya Kiosks were associated with enhanced access to MNH care, with significant improvements observed in newborn examination within 2 days after delivery. More women sought care from private clinics at end-line compared to baseline, indicating potential for private sector in supporting health service delivery gaps in under-served settings.

## Background

In the assessment of progress towards the fifth millennium development goal, there were an estimated 216 global maternal deaths per 100,000 live births in 2015, a decline of 44% from 1990 [1]. Kenya showed insufficient progress in this indicator with 17% decline in maternal mortality ratio from 490 in 1990 [1]. The 2014 Kenya Demographic and Health Survey (KDHS) reported 362 maternal deaths per 100,000 live births for the seven-year period preceding the survey and indicated that this was not significant from the ratios reported in 2003 and 2008–09 KDHS [2]. Maternal health indicators vary across the country with about 15 counties accounting for 98.7% of all maternal deaths [3]. Homa Bay is one of the counties with the highest maternal deaths; one mother dies for every 171 who undergo successful deliveries and one newborn succumbs for every 37 born [2]. Homa Bay County bears the largest burden of HIV in the country with a prevalence of 26% and is in the malaria endemic zone with intense malaria transmission throughout the year which further complicate survival for the rural poor of Homa Bay, with women and under-5 s being the most vulnerable [4, 5]. Documented challenges to maternal and child health in the country arise from an interplay of social, cultural, economic, and logistical barriers, coupled with a high fertility rate [6]. Most of the MNH related deaths occur due to delay in accessing care during childbirth and inadequate utilization of antenatal and post-natal care services. The nature of livelihood for the rural poor of Homa Bay makes it difficult to create time for routine MNH care or for timely skilled obstetric care access.

Addressing barriers to access to MNH, and other primary healthcare services is pivotal in reversing the trend of high maternal, newborn and under-5 morbidity and mortality [7]. However, facilitating access to quality health services in under-served populations remains a challenge on account of difficulties in defining sustainable strategies. Public providers are often stretched and have limited reach. Private health enterprises may support access. In urban settings, up to 50% of the population access care from private providers [8]. However, private providers invest little in rural communities because of less entrepreneurial viability: rural settings are often not as densely populated as urban settings and mostly constitute populations at the bottom of the pyramid. Many social interventions have attempted to address the gaps in access but often fall short on sustainability, providing sporadic supply of health services and commodities [9]. Innovating sustainable rural healthcare service is critical in facilitating optimal MNH, primary health services access, and universal healthcare in general. To live up to this defined need, we developed and implemented a rural health-care access model of community medical centres: the Ubuntu-Afya Kiosks.

Ubuntu-Afya Kiosks define a network of community medical centres, unique in their co-ownership model with beneficiary communities, often engaged as community self-help groups, who contribute to the development of the required infrastructure. These community groups are involved in overseeing the operations of the medical centres, essentially enhancing social responsibility, supporting business endurance, securing market loyalty and helping to navigate socio-political challenges. The medical service is developed alongside supplementary enterprises, usually community savings and credit schemes or motorbike taxi services, with the begotten revenue helping to cross-subsidize the cost of care and providing direct value to the community members. To complement this business model, the kiosks often partner with county governments, leveraging human resource and universal health commodity support, essentially enhancing sustainability of the interventions. We have implemented this people-public-private partnership (PPPP) approach in 16 sites in Homa Bay, being part of 25 in our network in mostly rural Kenya.

We examined the effect of this model of community medical centres on MNH access in Homa Bay, over a 2 year period of intervention.

## Methods

### Study objective and design

We evaluated whether the Ubuntu-Afya Kiosks had an effect on: the proportion of expectant mothers who attend at least four antenatal care (ANC) visits; the proportion of women who deliver under a skilled birth attendant; and the proportion of newborn children who undergo post-natal check within the first 2 days of delivery. We interrogated these questions using a non-randomized before-after study design.

### Study setting

Sixteen Ubuntu-Afya Kiosks were set up in rural Homa Bay between March and December 2016, all operational to-date. In this evaluation, ten community health units (CHUs) served by 10 community medical centres were conveniently sampled from three sub-counties: Rachuonyo North, Kabondo Kasipul, and Mbita. Homa Bay has an approximate population of 1,177,181: 266,946 are women of child bearing age (15–49 Years) and 214,647 thousand are children under-5 [10]. The proportion of births assisted by a skilled provider, proportion of women having 4 or more ANC visits, and the proportion of births with a postnatal checkup in the first 2 days after birth were documented as 60, 59 and 41% by the 2014 KDHS survey report. The maternal mortality rate is 583/100,000 [11], and the infant mortality is 77/1000. As at 2016, 226 public and private health facilities were documented as duly registered [12], representing a population to health facility ratio of 2.1 per 10,000 [13].

### Study population

For this evaluation, women of child bearing age (15–49 Years) with children aged 2 years or below or who had been expectant within the preceding 2 years were recruited. Women and children who had just moved into the catchment CHUs within the preceding 6 months were excluded from the study.

### Study period

Set up of the community medical centres was complete in December 2016. A baseline survey was conducted in June 2016. An end-line survey was conducted in May 2018.

### Study procedure

Recruitment of study subjects was done through sampling of consecutive households in CHU clusters. Interviewers visited households within the selected CHUs and administered a standardized questionnaire to the eligible participants (Additional file 1). The questionnaire was deployed using Open Data Kit (ODK) tool and included individual respondent characteristics and key questions on setting of last delivery; number of visits to a health facility; and examination of the newborn post-delivery in the previous pregnancy. Trained data collectors who understood the local language administered the standardized questionnaire with support from community health volunteers employed by the county under the community health strategy unit. The questionnaire was tested prior to the baseline assessment and revised to assure validity and reliability. Regular spot checks were conducted by the project team to verify authenticity of the data collected. Completed questionnaires were checked for missing information, inconsistencies, and wrong skip patterns which were rectified before submission for data entry. Key informant interviews were conducted at end-line to give context to the findings of the household surveys. The key informants were members of sub-county health management team, in particular: sub-county medical officers of health; District AIDS/STI coordinator; public health nurses; health promotion officers; and clinical officers.

### Data management and analysis

The study was designed to detect at least a 10% change in the proportion of women who deliver under a skilled birth attendant against a population prevalence of 55%, with a desired power of 80% and 5% level of significance. The estimated required number of respondents for each survey was 396 women. Evaluation of the effect of Ubuntu-Afya kiosks was based on three core outcome indicators of access to MNH services: (1) percent of women who attended four ANC visits during previous pregnancy; (2) percent of women who delivered under skilled birth attendant; and (3) percent of newborn children examined within 2 days after delivery. The study database excluded personal identifiers and was maintained in a central server with restricted access. We analysed the data using Intercooled STATA version 13.0 (StataCorp 4905 Lakeway Drive College Station, TX 77845 USA).

Background characteristics (marital status, level of education, and source of income) were analysed using Fisher’s exact test to examine the hypothesis of independence between respondent characteristic at baseline and end-line. T-test was used to test the hypothesis of no difference in mean age of the respondents during baseline survey compared to end-line. Descriptive statistics were used to summarize proportion in antenatal care, skilled delivery, and postnatal care at baseline and at end-line. Based on the sampling design, individuals in a select CHU were considered likely to bear more similarity to each other compared to individuals in other CHUs. Clustering at the level of CHUs was therefore assumed and STATA ‘svy’ commands were used to calculate Confidence Intervals (CIs) for the proportions. Estimated proportions; *p*-values of the hypothesis of no difference between baseline and end-line; and respective 95% CIs where the CIs indicate the range within which there is 95% confidence where the true value for the estimate lies, are presented.

### Ethical considerations

The study was approved by the African Medical Research Foundation Ethical Review Committee (AMREF-P235–2016). Written informed consent was sought from study participants during both surveys, and parental consent and study participant’s assent were sought for participants below 16 years of age. Participants were made aware that their participation was of free will and that they could withdraw from the study should they change their minds about participation. Confidentiality was maintained during the survey and study data was anonymised and maintained in servers accessible only by the principal investigators.

## Results

Four hundred and forty one women were interviewed at baseline and 408 women were interviewed at end-line.

The median age of the respondents was 25 (IQR 20–30) and 25 (IQR 21–30) years at baseline and end-line respectively. Most of the respondents were married or living with their spouses at the time of the survey (Table 1). Majority of the respondents were either unemployed or in informal employment: this was similar between baseline and end-line. Women who were sampled at end-line were more likely to be uneducated. For approximately two-thirds of the respondents at end-line, the age of last child was 1 year and below, implying that this group would most likely have experienced the services of Ubuntu-Afya kiosks during delivery of last child (Table 1).

**Table 1:** Characteristics of the respondents

Characteristic	Baseline % (n)	End-line % (n)	*P*-value
Mean age of respondents (in years)	26 (441)	26 (408)	0.788
Proportion by marital status:			
Currently married/living together	84	80	0.271
Single	10	14	
Separated/Divorced	1	2	
Widowed	5	4	
Proportion by main source of livelihood:			
Unemployed	42	42	1.000
Farming/Agriculture	13	13	
Informal employment	37	37	
Formal employment	8	8	
Proportion by highest level of education:			
No formal education	4	25	0.000
Primary school	78	55	
Secondary school	15	14	
College/University/Tertiary institution	3	6	
Proportion by age of last child:			
0 to 5 months	34	27	0.000
6 months to 1 year	46	36	
2 years to 5 years	20	37	

ANC attendance for previous pregnancy was significantly higher (99%) at end-line compared to baseline (81%). However, despite the high ANC attendance, only about seven out of ten mothers attended the recommended four ANC visits during the last completed pregnancy, and this was higher at end-line compared to baseline (Table 2).

**Table 2:** MNH indicators at baseline and end-line

Indicators	Baseline % (95% CI)	N	End-line % (95% CI)	N	*P*-value
Proportion of women who attended ANC during previous pregnancy	81 (78, 84)	441	99 (97, 100)	341	0.000
Proportion of women who attended four ANC visits during previous pregnancy	64 (59, 69)	358	71 (65, 76)	338	0.054
Proportion of women who delivered under skilled birth attendant	85 (80, 89)	364	90 (84, 94)	341	0.144
Proportion of mothers and newborns reviewed within 2 days of delivery.	74 (63,77)	364	92 (84,94)	341	0.000

The proportion of women who delivered under a skilled birth attendant (defined as doctors, nurses, or midwives in the Kenya health sector indicator manual) was higher at end-line (90%) compared to baseline (85%). There was an increase in the proportion of women who had their newborns examined within 1 day after delivery from 71% at baseline to 90% at end-line. Cumulatively, at end-line, 92% of the mothers had their newborn examined within 2 days compared to 74% at baseline (Table 2).

**Fig. 1:**
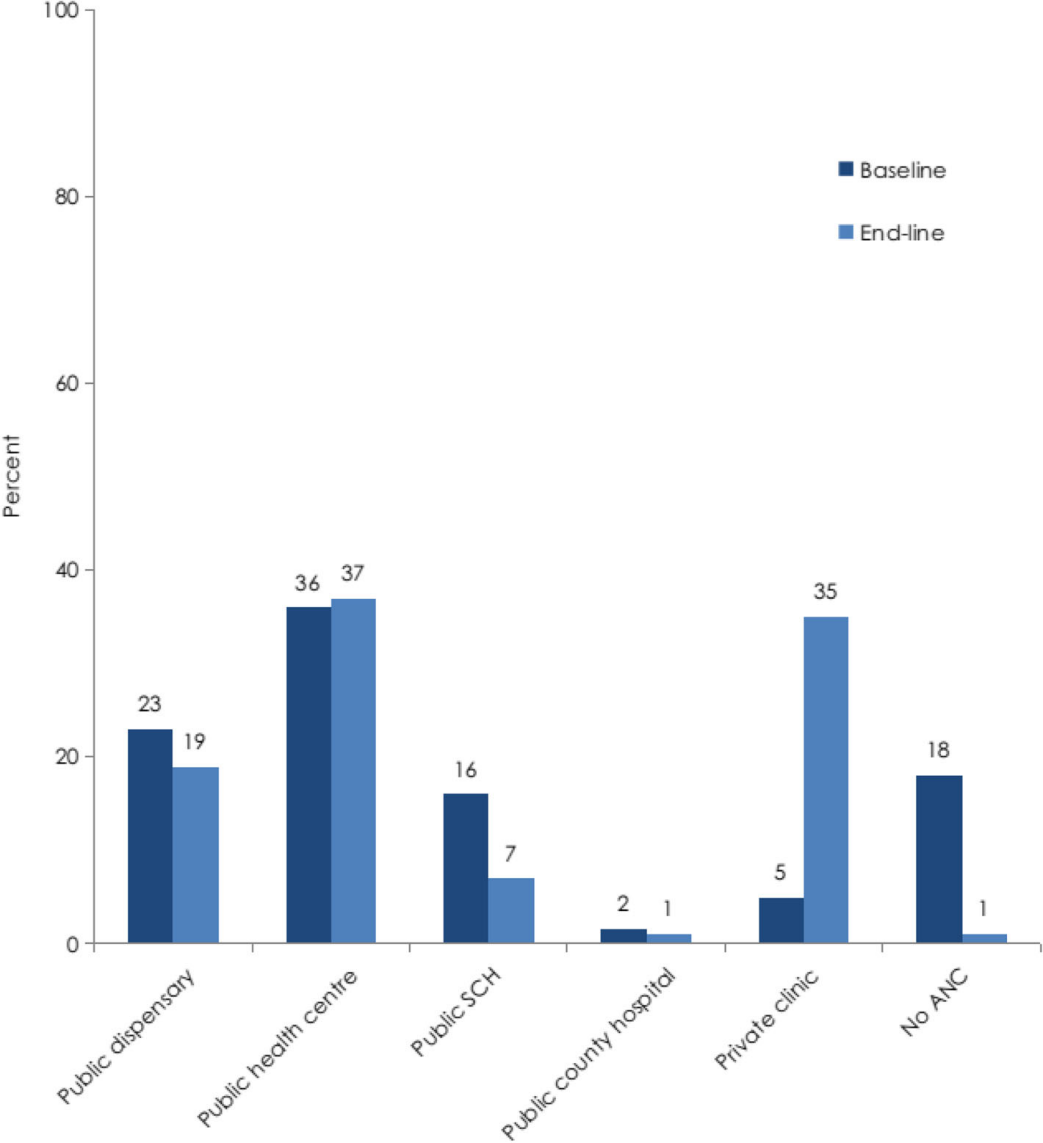
Proportion of women by place of ANC visit during previous pregnancy

More mothers at end-line sought MNH services from private clinics compared to baseline (Fig. 1).Seventy-five percent (86 of 114) of the respondents who had been attended to in a private facility at end-line indicated that they had been attended to at an Ubuntu-Afya Kiosk. Conversely, there was a significant reduction at endline in the proportion of mothers who sought care from county and sub-county hospitals.

Country-wide and county-wide MNH access indicators obtained from routine health information system were low over the study period (Table 3).

**Table 3:** MNH indicators corresponding to baseline and end-line periods (Data Source: DHIS2 RMNCH Scorecard provided on 03 December 2018)

Indicators	Homa Bay County		Kenya	
	June 2016	May 2018	June 2016	May 2018
Proportion of pregnant women who attended at least one ANC during pregnancy	84	90	81	90
Proportion of pregnant women attending at least four ANC visits during pregnancy	53	47	50	55
Delivery by skilled birth attendant	66	60	63	68
Proportion of newborn children examined within 2 days after delivery (post-natal care attendance coverage)	60	80	62	70

County government key informant interviewees acknowledged that implementation of Ubuntu-Afya had brought services closer to hard-to-reach communities with specific improvements in service utilization noted in immunization coverage and reduction in defaulter rates in immunization. In consideration of this role, the Homa Bay County Government took up support and operations of 7 community medical centres, essentially initiating a public-private partnership to support sustainability of the interventions.

## Discussion

We observed increased access to MNH services at end-line compared to baseline. There was a remarkable increase in the number of newborns who presented for post-natal examination in the first day after delivery, from 71% at baseline to 90% at end-line. There was a slight change in the proportion of women who attened four ANC visits, from 64 to 71%. In overall, at least one ANC attendance was 99% at end-line compared to 81% at baseline. Skilled delivery prevalence was impressively high even at baseline: nonetheless, an increase from 85 to 90% was observed.

More mothers sought care from private clinics at end-line than at baseline, with most respondents indicating they had accessed care at an Ubuntu-Afya centre. The corresponding reduction in proportion of mothers seeking care from the sub-county and county hospitals suggests that interventions to improve access to primary healthcare could reduce over-burdening of secondary health services and support improved MNH outcomes. The evaluation period coincided with a prolonged health worker industrial action which affected access to MNH and other primary healthcare services in public health facilities in the country. County-wide MNH indicators over the study period were lower in 2017 compared to 2016: skilled delivery rates dropped from 61% in 2016 to 49% in 2017, and 4th ANC access was at 43 in 2017 compared to 52 in 2016 [13]. Improved MNH indicators in our settings of interventions showcases the complementary role that could be played by innovative private health-care models in improving MNH access in poor under-served settings. The kiosks helped to address the delays associated with poor outcome in MNH care: essentially taking care to mothers and their newborns, rather than pushing mothers to access care in traditionally designed provider setups.

The greatest effect of our intervention was on access to post-natal care for newborns, perhaps suggesting a gap that is yet to be addressed by public health providers and the universal healthcare programmes. Studies in similar setups indicate progress in other MNH access indicators but stagnation in post-natal care coverage, suggesting the need for deliberate intervention [14]. It is likely that the kiosks made for convenient access locations for post-natal mothers and their newborns. There was significant effect on fourth ANC visits, although not congruent to the improvement observed with at least one ANC visits. This provides a pointer to the health seeking behaviour of the target population who likely access ANC care in advanced stages of pregnancy, not allowing for subsequent and optimal ANC visits. There is an opportunity for strategic targeting of the gestation at first antenatal care clinic so as to promote optimal care.

There is a role for innovative non-public health enterprise to complement public health investment to facilitate access to MNH and other primary healthcare services. However, there is very little literature on the effectiveness of private and other non-public community clinics in facilitating rural access to MNH and other primary healthcare services. This could possibly be as a result of the paucity of interventions by private sector players in rural settings on account of perceived non-viability of such initiatives. A review of primary health interventions in sub-Saharan Africa indicates the role of community ownership and mobilization as crucial facilitators for the sustainability of these interventions [9]. Community co-ownership is part of the make-up design of Ubuntu-Afya Kiosks and likely contributes to their sustainability to-date.

Because of limited reach by public health facilities and the lack of sustainability in programmatic interventions, rural settings fail to benefit from the potential efficiencies of private health enterprise and the complementarity in scale of mixed public and private interventions as has been observed in urban settings. Up to 50% of the Kenyan population accesses care from private, NGOs and faith-based service providers, but this is mostly the case for urban settings [15]. A significant proportion of the population, including rural communities, have to pay for service from out-of-pocket even when they are accessing service from the public health facilities [8]. About 27% of rural mothers access ANC care from private providers in rural Kenya [8]. The potential for private health enterprise as a facilitator of rural MNH access is therefore established.

In this study, the county government partnered with the investigators to secure sustainability of the centres and enhance their relevance to the target communities. As at the time of publication, seven of the kiosks were due for gazettement, essentially securing government human resource and commodity support. This public-private relationship showcases the possibilities in using PPPs to support primary healthcare access. Most PPPs in healthcare have focussed on higher levels of care and on equipment supply. There is room for innovation and policy development to support similar relationships to help governments scale their reach to their citizenry. By implementing a National Health Insurance Fund (NHIF) driving access to care from both public and private providers, the Kenyan government is essentially promoting PPPs in facilitating access to care. Deliberate strategies to increase NHIF coverage should be explored. The Kenya Government’s “Linda Mama” initiative is a laudable effort that will likely promote investment by private healthcare providers in rural settings.

In our study, recall error could have been a source of bias because respondents may or may not remember the number of MNH visits. Contextual information highlighted the challenge with influencing timing and number of ANC visits, with specific mention of delayed visits. The observed high prevalence in skilled delivery rates could also be on account of selection bias: those who delivered at home may have suffered complications or mortality and were therefore not available to provide responses, grossly over-estimating the proportion of those who delivered at health facilities. Notably, data from the routine health information indicates decreased skilled care delivery in the county from 61% in 2016 to 53% in 2017 due to the prolonged industrial action by health workers.

## Conclusion

Ubuntu-Afya kiosks were associated with enhanced MNH access, with the most significant improvement being observed in PNC review within 2 days of delivery. There was improved uptake of skilled delivery and ANC care in the target communities. More women sought care from private clinics, mostly Ubuntu-Afya Kiosks, at end-line indicating the opportunity for innovative private sector care models to complement government reach.

## Supplementary information

**Additional file 1.** Survey questionnaire used at baseline and endline.

## Data Availability

The data that support the findings in this paper are available from the lead author upon reasonable request and with permission from Afya Research Africa.

## Abbreviations

ANC: Antenatal care
CHUs: Community health units
CIs: Confidence intervals
HIV: Human immunodeficiency virus
KDHS: Kenya Demographic and Health Survey
MNH: Maternal and newborn health
NHIF: National Health Insurance Fund
PPPs: Public-private partnerships
