# Supplementary material for: Expanding access to maternal, newborn and primary healthcare services through private-community-government partnership clinic models in rural Kenya: the Ubuntu-Afya kiosk model
Source: BMC Health Serv Res. 2019 Nov 29;19:914. doi: 10.1186/s12913-019-4759-9 (PMC6884755; doi:10.1186/s12913-019-4759-9)
Supplement: Supplementary file 1 — Additional file 1. Survey questionnaire used at baseline and endline. [file 12913_2019_4759_MOESM1_ESM.doc]

**Appendix II: Questionnaire**

**QUESTIONNAIRE – UBUNTU AFYA KIOSK PROJECT HOMABAY**

Date: ___________________________

Time: _____________________________

County: _____________________________

Sub-county: ___________________________

Community Health Unit: ___________________________

Household number/Code: _________________

Interviewee code: __________________________

**1.0 Demographic Information**

1.1 Age of respondent

1.2 What is your religion?

1. Christian
2. Muslim
3. Traditional
4. Hindu
5. Other

1.3 What is the highest education level that you have completed?

1. Primary
2. Secondary/high school
3. College/University/Tertiary institution
4. Other

1.4 What is your marital status?

1. Currently married/living together as married
2. Separated/divorced
3. Widowed
4. Other

1.5 What is your main source of livelihood currently?

1. Formal business
2. Informal business
3. Formal salaried
4. Informal salaried
5. Formal casual
6. Informal casual
7. Farming/Agriculture
8. Unemployed
9. Other

**2.0 Current Antenatal care**

2.0. Are you currently pregnant?

1. Yes

2. No **[*If not pregnant skip to section 3.0*]**

2.1 How many weeks pregnant are you?

2.2 Are you visiting any facility/person for antenatal care for this pregnancy?

1. Yes ***[Skip to Q2.4]***
2. No

2.3 If No, why not?

1. Cost is too high
2. Facility not open
3. Distance of the facility/doctor from home
4. Am not aware of available health facilities/services
5. Poor quality service
6. No female provider
7. Husband/family didn’t want me to
8. Cultural preference
9. Religious preference
10. Am planning to start visiting one
11. Other

**{Then skip to section 2}**

2.4 If yes, which facility are you visiting?

1. GoK dispensary
2. GoK Health centre
3. GoK Sub-county/district hospital
4. GoK County/provincial hospital
5. Private maternity and nursing home
6. Private clinics
7. Private hospital
8. Traditional Birth attendant premises
9. Other

2.5 Who attends to you during your antenatal care visits?

1. Doctor
2. Nurse
3. Midwife
4. Community health worker
5. Traditional birth attendant
6. Don’t know
7. Other

2.6 How many antenatal care visits have you gone for in the above named facility?

2.7 During your antenatal care visits what were the services that you received?

1. Weight checked
2. Blood pressure
3. Urine tested
4. Blood tested
5. Stool tested
6. Ultrasound done
7. HIV test done
8. Iron pills given
9. Malaria pills given
10. Don’t know/don’t remember [***Select only if none of the above is selected***]
11. Others

2.8 During you’re the antenatal visits, what information or counseling did you receive?

1. Pregnancy test
2. Place of delivery
3. Health during pregnancy
4. Nutrition in pregnancy
5. HIV/AIDS
6. Breastfeeding
7. Infant feeding
8. Others

**3.0 Previous Antenatal care**

3.1 How old is your last child?

1. Record age of last child ___________________
2. If last child is deceased, record age of child at death __________________
3. No other child and pregnant with first child **[Skip to section 4.0]**

3.2 When you were pregnant with your last child, did you visit any health facility/anybody for antenatal care?

1. Yes
2. No ***[Skip to Q3.4]***

3.3 If yes, which facility did you visit?

1. Home/community
2. GoK dispensary
3. GoK Health center
4. GoK Sub-county/district hospital
5. GoK County/provincial hospital
6. Private maternity and nursing home
7. Private clinics
8. Private hospital
9. Other ______________________________

***[Then skip to Q3.5]***

3.4 If No, why not?

1. Cost was too high
2. Facility not open
3. Distance of the facility/doctor from home
4. Wasn’t aware of available health facilities/services
5. Poor quality service
6. No female provider
7. Husband/family didn’t want me to
8. Cultural preference
9. Religious preference
10. Other

***[Then skip to 3.8]***

3.5 Who attended/gave you antenatal care during the last pregnancy?

1. Doctor
2. Nurse
3. Midwife
4. Community health worker
5. Traditional birth attendant
6. Don’t know/cant remember
7. Other

3.6 In your last pregnancy, during your antenatal care visits what were the services that you received?

1. Weight checked
2. Blood pressure
3. Urine tested
4. Blood tested
5. Stool tested
6. Ultrasound done
7. HIV test done
8. Iron pills given
9. Malaria pills given
10. Don’t know/don’t remember
11. Others

3.7 In your last pregnancy, during your the antenatal visits, what information or counseling did you receive? ***[Select all that are mentioned]***

1. Pregnancy test
2. Place of delivery
3. Health during pregnancy
4. Nutrition in pregnancy
5. HIV/AIDS
6. Breastfeeding
7. Infant feeding
8. Others

3.8 Where did you deliver your last child?

1. Home/community [***If delivered at home, skip to 3.10]***
2. GoK dispensary
3. GoK Health center
4. GoK Sub-county/district hospital
5. GoK County/provincial hospital
6. Private maternity and nursing home
7. Private clinics
8. Private hospital
9. Other

3.9 During delivery of your last child who helped you deliver/who facilitated the delivery?

1. Doctor
2. Nurse
3. Midwife
4. Community health worker
5. Traditional birth attendant
6. Don’t know/cant remember
7. Other

***[Then skip to Q3.12]***

3.10 If delivered at home/community, what was the reason you didn’t deliver at a health facility?

1. Facility not open
2. Cost was too high
3. Distance of the facility from home
4. Wasn’t aware of available health facilities
5. Poor quality service
6. No female provider
7. Husband/family didn’t want me to deliver at a health facility
8. Delivery was unexpected
9. Cultural preference
10. Religious preference
11. Other

3.11 If delivered at home/community, who helped you deliver/who facilitated the delivery?

1. Community health worker
2. Traditional birth attendant
3. Don’t know/cant remember
4. Others

3.12 After you delivered your child after how many days/hours did was the newborn examined by a care giver?

1. Within one day
2. Within two days
3. After 3 days
4. Other ___________

3.13 Who examined the newborn after delivery?

1. Doctor
2. Nurse
3. Midwife
4. Community health worker
5. Traditional birth attendant
6. Don’t know/cant remember
7. Other _________________________

**4.0 Post natal care**

4.1 Did you go for postnatal care after the delivery of your last child?

1. Yes
2. No ***[Skip to Q4.5]***

4.2 If yes, how many postnatal visits did you go for?

4.3 Where did you receive the postnatal care?

1. Home/community
2. GoK dispensary
3. GoK Health center
4. GoK Sub-county/district hospital
5. GoK County/provincial hospital
6. Private maternity and nursing home
7. Private clinics
8. Private hospital
9. Other

4.4 Who attend to you during your last postnatal care?

1. Doctor
2. Nurse
3. Midwife
4. Community health worker
5. Traditional birth attendant
6. Don’t know/cant remember
7. Other

4.5 If no, what was the reason for not going for post-natal care?

1. I wasn’t feeling sick
2. Gave birth at home, so didn’t see need to go
3. The health centre was far from home
4. Didn’t have money to go to the health centre
5. Had a bad experience during antenatal care / labor / delivery
6. Others (Specify):

**5.0 Previous pregnancy outcome/complications**

5.1 Were there any complications during your last pregnancy or after delivery?

1. Yes
2. No ***[If no, skip to section 6]***

5.2 If yes, what were the complications?

1. High blood pressure
2. Anemia
3. Bleeding/spotting
4. Severe nausea/vomiting
5. Malaria
6. Fainting
7. Varicose veins
8. Swollen legs
9. Fever
10. Newborn death ***[Skip to section 6]***
11. Other

5.3 How were the complications managed?

5.4 Do you feel/think the complications were managed appropriately?

1. Yes
2. No

5.5 Have you/your child recovered from the complications?

1. Yes
2. No

5.6 If no, what persists?

**6.0 Digital capture**

6.1 Do you have an antenatal card/book?

1. Yes
2. No (If no, end interview and thank respondent)

6.2 If yes, to above, can I see the clinic card and take photos of page 5, 6, 11 and 13?

1. Accepts photos of card to be taken
2. Declines for photos to be taken

Thank the respondent and end the interview.

6.0 Record the GPS coordinate within the venue/homestead where interview was done.

**Interview result:**

01: Completed

02: Partially completed

**Name of interviewer:**
